# Automatic segmentation and classification of breast lesions through identification of informative multiparametric PET/MRI features

**DOI:** 10.1186/s41747-019-0096-3

**Published:** 2019-04-27

**Authors:** Wolf-Dieter Vogl, Katja Pinker, Thomas H. Helbich, Hubert Bickel, Günther Grabner, Wolfgang Bogner, Stephan Gruber, Zsuzsanna Bago-Horvath, Peter Dubsky, Georg Langs

**Affiliations:** 1 Computational Imaging Research Laboratory, Department of Biomedical Imaging and Image-guided Therapy, Medical University Vienna, Waehringer Guertel 18-20, 1090 Vienna, Austria; 2 Division of Molecular and Gender Imaging, Department of Biomedical Imaging and Image-guided Therapy, Medical University Vienna, 1090 Vienna, Austria; 3 Department of Radiology, Breast Imaging Service, Memorial Sloan Kettering Cancer Center, New York, NY 10065 USA; 4 MR Center of Excellence, Department of Biomedical Imaging and Image-guided Therapy, Medical University Vienna, 1090 Vienna, Austria; 5 Department of Pathology, Medical University Vienna, 1090 Vienna, Austria; 6 Department of Surgery, Medical University Vienna, 1090 Vienna, Austria; 7 Department of Radiologic Technology, Carinthia University of Applied Sciences, Klagenfurt, Austria

**Keywords:** Diagnosis (computer-assisted), Breast neoplasms, Magnetic resonance imaging, Machine learning, Positron-emission tomography

## Abstract

**Background:**

Multiparametric positron emission tomography/magnetic resonance imaging (mpPET/MRI) shows clinical potential for detection and classification of breast lesions. Yet, the contribution of features for computer-aided segmentation and diagnosis (CAD) need to be better understood. We proposed a data-driven machine learning approach for a CAD system combining dynamic contrast-enhanced (DCE)-MRI, diffusion-weighted imaging (DWI), and ^18^F-fluorodeoxyglucose (^18^F-FDG)-PET.

**Methods:**

The CAD incorporated a random forest (RF) classifier combined with mpPET/MRI intensity-based features for lesion segmentation and shape features, kinetic and spatio-temporal texture features, for lesion classification. The CAD pipeline detected and segmented suspicious regions and classified lesions as benign or malignant. The inherent feature selection method of RF and alternatively the minimum-redundancy-maximum-relevance feature ranking method were used.

**Results:**

In 34 patients, we report a detection rate of 10/12 (83.3%) and 22/22 (100%) for benign and malignant lesions, respectively, a Dice similarity coefficient of 0.665 for segmentation, and a classification performance with an area under the curve at receiver operating characteristics analysis of 0.978, a sensitivity of 0.946, and a specificity of 0.936. Segmentation but not classification performance of DCE-MRI improved with information from DWI and FDG-PET. Feature ranking revealed that kinetic and spatio-temporal texture features had the highest contribution for lesion classification. ^18^F-FDG-PET and morphologic features were less predictive.

**Conclusion:**

Our CAD enables the assessment of the relevance of mpPET/MRI features on segmentation and classification accuracy. It may aid as a novel computational tool for exploring different modalities/features and their contributions for the detection and classification of breast lesions.

**Electronic supplementary material:**

The online version of this article (10.1186/s41747-019-0096-3) contains supplementary material, which is available to authorized users.

## Key points

- The positron emission tomography/magnetic resonance imaging (PET/MRI) computer-aided segmentation and diagnosis (CAD) system automatically detects, segments, and classifies breast lesions.
- Automatic lesion segmentation was accurate and improved with information from all modalities.
- A small number of features mainly from dynamic contrast-enhanced MRI achieves high classification accuracies.
- The PET/MRI-CAD system allows exploring the value of different imaging modalities and features.

## Background

**Fig. 1:**
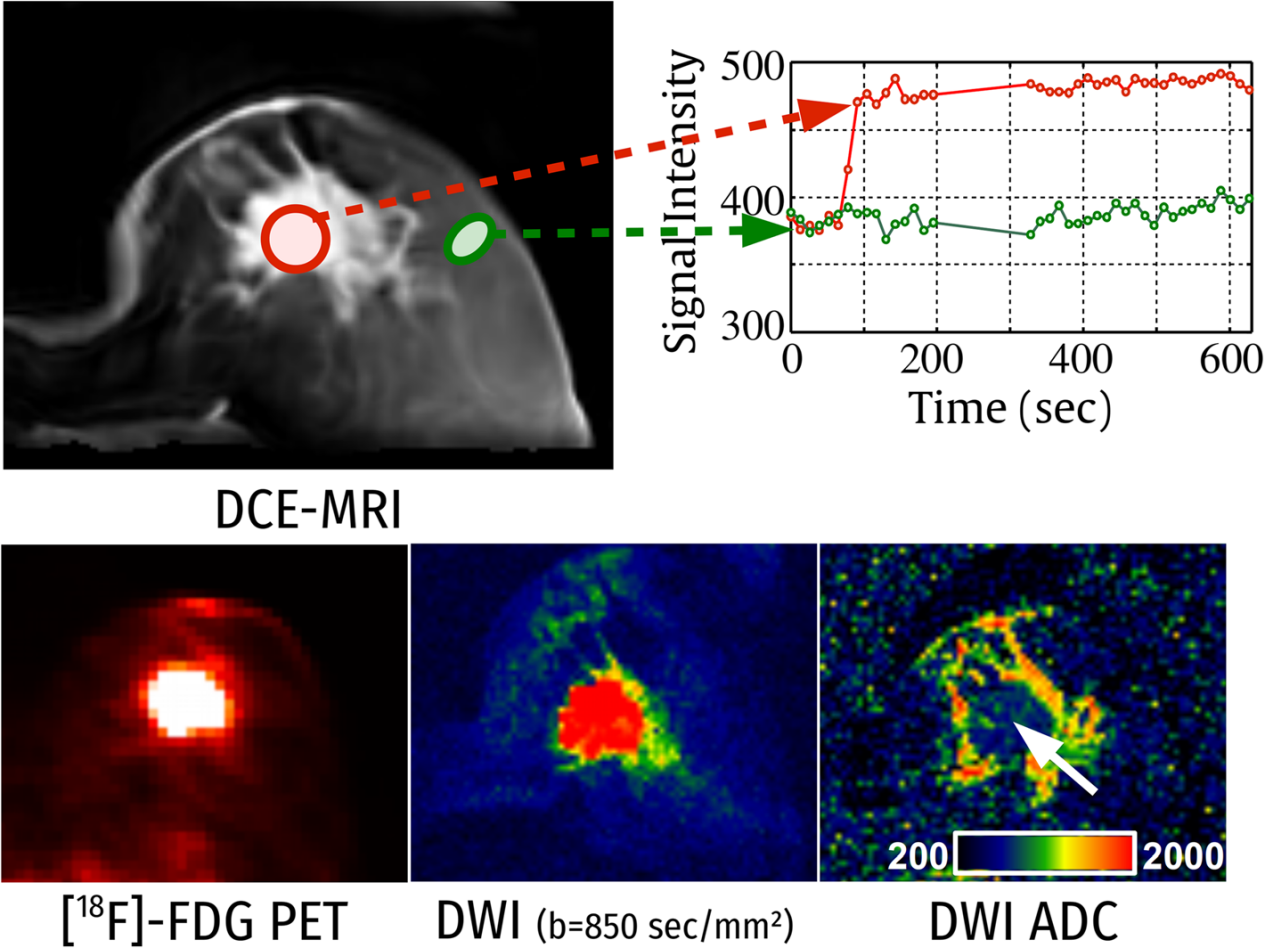
Image modalities covering the lesion. Top: DCE-MRI time-signal intensity curve extracted from an ROI within the lesion (red) and from normal tissue (green), illustrating the contrast enhancement within the lesion. Bottom from left to right: ^18^F-FDG-PET, DWI, and ADC map. Note the decreased ADC values in the lesion area (white arrow)

Breast cancer is the most common cancer and the second most common cause of mortality from cancer in women [1]. Early detection and precise diagnosis are important for effective treatment [2], and breast imaging plays a pivotal role in the detection, characterisation, and staging of breast cancer. Recently, multimodal, multiparametric imaging (mpI) including dynamic contrast-enhanced magnetic resonance imaging (DCE-MRI), diffusion-weighted imaging (DWI), and positron emission tomography (PET) has been investigated for an improved differentiation of benign and malignant breast lesions [3]. Such imaging constitutes complex protocols but is promising for a more comprehensive measurement of morphology (MRI), neoangiogenesis (DCE-MRI), tumour metabolism (PET), and microstructure (DWI) in cancerous and benign tissue [3] (Fig. 1).

Due to the increased complexity of the information captured by mpI, computational approaches that enable the quantitative assessment of multivariate measurements have been gaining relevance. Recently, computer-aided detection and diagnosis systems have been proposed to reduce inter- and intra-reader variability and to aid radiologists in the detection and diagnosis of breast cancer [4]. These systems are able to analyse large amounts of imaging data in a short time, detect and visualise complex correlations and patterns, and provide objective and repeatable measurements [5] to increase the accuracy of diagnosis [6]. Computer-aided detection (CADe) systems assist radiologists in localising suspicious regions in medical images, whereas computer-aided diagnosis (CADx) systems support the radiologist in the diagnosis of suspicious regions by providing and analysing information extracted from these regions [7]. These systems show potential to be advantageous in the current clinical scenario [7] where despite guidelines for DCE-MRI, such as the Breast Imaging-Reporting and Data System (BI-RADS®) MRI lexicon [8], inter- and intra-reader variability remains an issue and the human analysis of complex relationships observed in images and the underlying disease remains limited [9].

As yet, the information provided by individual imaging techniques as part of mpI remains poorly understood. To identify the diagnostically relevant parameters captured across DCE-MRI, DWI, and ^18^F-fluorodeoxyglucose (^18^F-FDG)-PET, we propose a novel automated data-driven approach: a combined breast lesion segmentation and classification system for mpI data where the system automatically identifies the information in the imaging data that contribute to an accurate segmentation and classification.

## Methods

### Patients

The data used in this retrospective analysis was acquired from an institutional review board-approved prospective, single-institution study [25]. All patients gave written informed consent. At the time of the prospective study, only prototypic PET/MRI scanners were in existence and these were not available at the study centre. Thus, 46 patients were included in this prospective study in which MRI and a combined computed tomography (CT)/^18^F-FDG-PET were acquired. All tumours were histopathologically verified. In our retrospective analysis, the CT image was used only as morphologic information for the registration and was purposely not part of segmentation and classification. After applying our automatic CT to MRI registration method, as described below, twelve patients had to be removed from analysis due to registration errors. All excluded cases were patients with large breasts that were considerably compressed, or deformed, in one of the modalities during image acquisition. Misalignments were detected visually by overlaying MRI and CT images. From the remaining 34 patients, 12 had benign lesions and 22 had malignant lesions (2 patients had multifocal or multicentric cancer). Characteristics of the lesions are listed in Table 1.

**Table 1:** Patient and breast lesion characteristics

Patients (*n* = 34) Age, years, mean ± SD (range)	51.36 ± 12.26 (24–78)
Lesions (*n* = 36) Diameter, cm, mean ± SD (range)	1.8 ± 1.1 (0.6–4.9)
Lesion type	
Malignant	24 (66.7%)
Invasive ductal carcinoma	19 (52.8%)
Invasive lobular carcinoma	3 (8.3%)
Ductal carcinoma *in situ*	2 (5.6%)
Benign	12 (33.3%)
Total	36 (100.0%)

*SD* standard deviation

### Image acquisition

Patients underwent 3T MRI (Tim Trio, Siemens, Erlangen, Germany) in prone position using a four-channel breast coil (InVivo, Orlando, FL, USA) and a combined whole-body PET/CT in-line system (Biograph 64 TruePoint®; Siemens, Erlangen, Germany) in prone position.

For DCE-MRI a split dynamics protocol that combined high-spatial and high-temporal resolution was used [11]. First, a high spatial resolution, pre-contrast coronal T1-weighted turbo three-dimensional fast low angle shot (FLASH) sequence with water-excitation and fat-suppression was acquired with matrix 320 × 320 × 120 and 1-mm isotropic voxel (DCE-MRI pre-contrast imaging, **I**_dce-pre_). Subsequently, a DCE coronal T1-weighted volumetric interpolated breath-hold-examination (VIBE) sequence with 17 acquisitions (13.2 s per acquisition) was acquired with matrix 192 × 192 × 72 mm and 1.7-mm isotropic voxel (DCE-MRI, **I**_dce_). Seventy-five seconds after the beginning of the sequence, gadoterate meglumine (Gd-DOTA, Dotarem®, Guerbet, Paris, France) was injected as a bolus at a dose of 0.1 mmol/kg at a rate of 4 mL/s and followed by a 20-mL saline flush at the same injection rate. Then, a FLASH sequence was acquired to capture the peak enhancement of lesions (DCE-MRI peak-contrast imaging, **I**_dce-peak_), followed by a VIBE sequence with the same parameters above described. Finally, a FLASH sequence with the same parameters above described was acquired (DCE-MRI post-contrast imaging, **I**_dce-post_*)* to depict delayed enhancement lesion morphology. DWI sequences were acquired in the same session, with *b* values of 50 and 850 s/mm^2^, resulting into two datasets, **I**_dwi b0_ and **I**_dwi b850_, as well as the derived apparent diffusion coefficient (ADC) mapping, **I**_adc_ [12] (matrix 172 × 86 × 24, pixel 2.09 × 2.09 mm, slice thickness 5.5 mm). ^18^F-FDG-PET (matrix 168 × 168 × 74, pixel 4 × 4 mm, slice thickness 3 mm) and CT images (matrix 512 × 512 × 74, pixel 1.37 × 1.37 mm, slice thickness 3 mm) of the thorax were acquired in a hybrid PET/CT scanner and were aligned by the scanner software.

### CAD pipeline

**Fig. 2:**
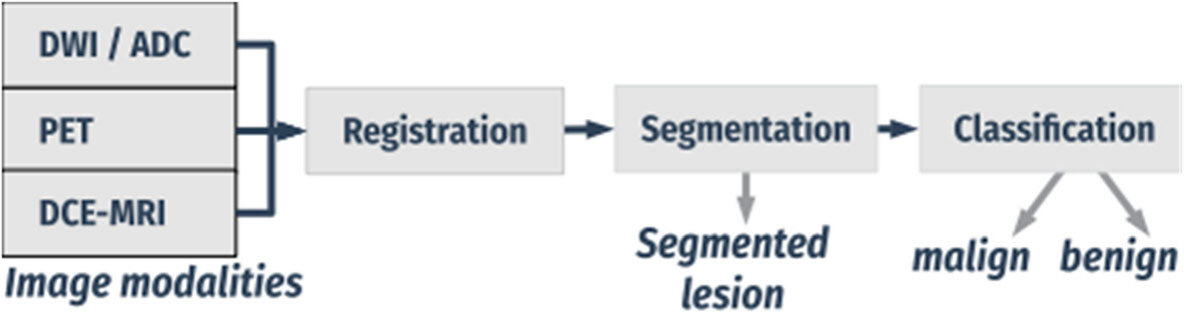
Overview of the CAD pipeline based on multimodal and mpI features

We developed a novel automated data-driven combined CADx system for mpI data with MRI and PET. The system enabled automatic detection and segmentation of potentially cancerous regions and classified lesions as benign or malignant. The algorithm first aligned multimodal breast imaging data from DCE-MRI, DWI, and ^18^F-FDG PET non-rigidly, and segmented the breast. Then, the system extracted local textural, kinetic, and intensity-based image features from the fused information and detected and classified lesions using a random forest (RF) classifier [10]. Figure 2 shows the overview of the proposed CAD pipeline.

### Alignment

**Fig. 3:**
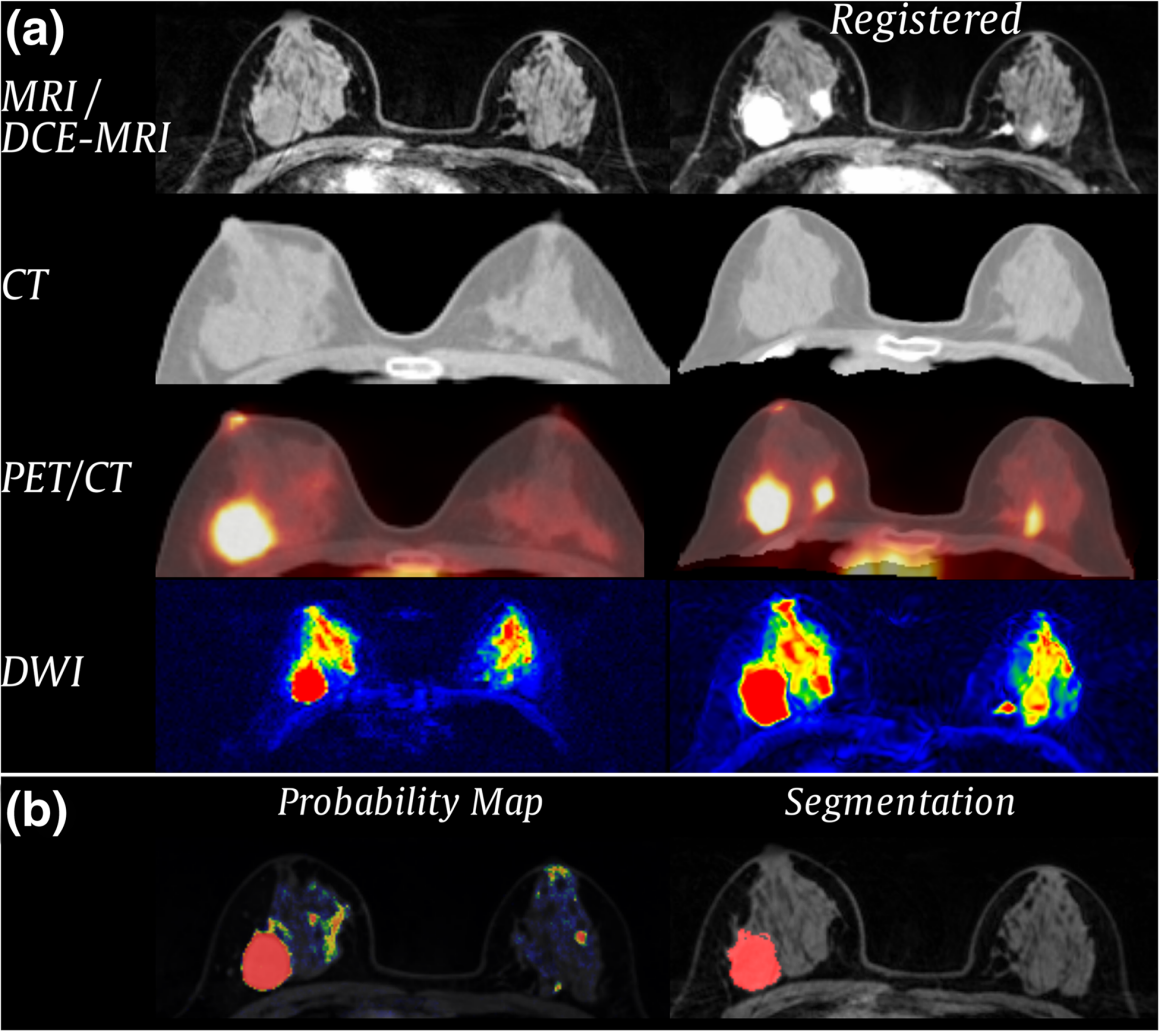
Results of the (**a**) registration and (**b**) segmentation process for one patient. **a** First row: Reference I_dce-pre_ and registered I_dce-post_. Second row: I_ct_ unregistered/registered. Third row: I_pet_ image unregistered/registered, fused with the corresponding CT image. Fourth row: I_dwi b0_ unregistered/registered. **b** Probability map obtained from voxel-wise classification overlaid on the MR pre-contrast image (left) and final segmentation after applying a threshold and post-processing (right)

To collect information at individual positions across modalities, all images were aligned to **I**_dce-pre_ serving as reference coordinate system. Images were registered with the software package Advanced Normalisation Tools (ANTs) [13] using an affine transformation with mutual information as the similarity metric, followed by a non-rigid deformation with symmetric normalisation (SyN) [13] and windowed normalised cross-correlation as a similarity metric (Fig. 3a). As **I**_pet_ does not provide morphologic information, we registered the corresponding CT image to **I**_dce-pre_ [14] and subsequently applied the obtained transformation on **I**_pet_.

### Lesion segmentation

We treated lesion segmentation as a voxel-wise classification problem, where a machine learning algorithm assigned a binary label 1 (lesion) or 0 (non-lesion) to each voxel based on imaging features extracted at that location. As ground truth for training and validation, we used manual expert radiologist (with 3 years of experience) annotations performed on the registered **I**_dce-peak_ or **I**_dce-post_, depending on where the lesion borders were better visible. Annotations were validated by a second expert radiologist with 9 years of experience.

All computations were restricted to the breast area, which was segmented using an intensity-based growing region algorithm [15]. All MRI intensity values were standardised to zero mean and unit standard-deviation estimated from the breast area on the pre-contrast images, **I**_dce-pre_ and **I**_dce_. We computed intensity features from all imaging data, from changes of the contrast over time and the summed up contrast in the DCE-MRI sequence as specified in Table 2.

**Table 2:** Features extracted for each voxel (**x**) within the breast (M)

Feature group	Description	Definition	Number of features per voxel
**f**_dce_	DCE-MRI intensity values for each frame of the DCE-MRI time series	**f**_dce_(**x**) ≔ {**I**_dce_(**x**, *i*), *j* ≤ *i* ≤ *j* + 25}, where *j* is the first frame with contrast-enhancement and *i* is the frame number in the DCE sequence	26
**f**_δdce_	Difference of DCE-MRI intensity values between two frames with distance 2	${\mathbf{f}}_{\updelta \mathrm{dce}}\left(\mathbf{x}\right):= \left\{\frac{{\mathbf{I}}_{\mathrm{dce}}\left(\mathbf{x},i+2\right)-{\mathbf{I}}_{\mathrm{dce}}\left(\mathbf{x},i\right)}{t_{i+2}-{t}_i}|j\le i\le j+25\right\}$, where *t*_*i*_ is the time point of acquisition of frame *i*	25
**f**_nsumdce_	Normalised sum of DCE-MRI intensities	${\mathbf{f}}_{\mathrm{nsumdce}}\left(\mathbf{x}\right):= \frac{{\mathbf{f}}_{\mathrm{sumdce}}\left(\mathbf{x}\right)}{\max_{y\in M}\left({\mathbf{f}}_{\mathrm{sumdce}}(y)\right)}$ where ${\mathbf{f}}_{\mathrm{sumdce}}\left(\mathbf{x}\right):= {\sum}_{i=j}^{j+25}{\mathbf{I}}_{\mathrm{dce}}\left(\mathbf{x},i\right)$	1
**f**_mri_	Intensity values for high-resolution MRI: **I**_dcepre_, **I**_dcepeak_, and **I**_dcepost_	**f**_mri_(**x**) ≔ {**I**_dcepre_(**x**), **I**_dcepeak_(**x**), **I**_dcepost_(**x**)}	3
**f**_δmri_	Difference in intensity values for high-resolution MR images	**f**_δmri_(**x**) ≔ {**I**_dcepost_(**x**) − **I**_dcepre_(**x**), **I**_dcepeak_(**x**) − **I**_dcepre_(**x**), **I**_dcepost_(**x**) − **I**_dcepeak_(**x**)}	3
**f**_dwi_	DWI intensity value	**f**_dwi_(**x**) ≔ {**I**_dwi *b*0_(**x**), **I**_dwi *b*850_(**x**), **I**_adc_(**x**)}	3
**f**_pet_	PET intensity value	**f**_pet_(**x**) ≔ {**I**_pet_(**x**)}	1

*DCE-MRI* dynamic contrast-enhanced magnetic resonance imaging, *DWI* diffusion-weighted imaging, *PET* positron emission tomography

An RF classifier model was trained on features extracted from 1000 randomly selected samples per class and patient. The trained model was then used to predict the segmentation label for a new patient who was not part of the training data set for each voxel *x* of the breast based on the computed features (Fig. 3b).

### Lesion classification

After segmentation, the lesion was classified as either benign or malignant based on features extracted per lesion. Intensity-based, kinetic, morphological, and textural features were considered to train a lesion class prediction model, and the obtained model was used to predict malignancy for lesions in the new patient who was not part of the training data set.

Intensity-based features were calculated from DCE-MRI, DWI ADC, and the ^18^F-FDG-PET map. We tackled the lesion inhomogeneities in the contrast enhancement of DCE-MRI by the method described by Chen et al. [16], where the signal-to-time curves within a lesion were clustered by the fuzzy c-means algorithm and the curve with highest contrast enhancement rate, the characteristic kinetic curve, was chosen for classification. We used the 25 time points beginning with contrast enhancement (*f*_lckc_) and the change over time (*f*_lδckc_) calculated by forward difference (four frames) as intensity features. Analogously, **I**_adc_ and **I**_pet_ intensities were partitioned into five clusters and the cluster centre with the lowest ADC value and the highest ^18^F-FDG uptake were used as features **f**_l-adc_ and **f**_l-pet_.

To capture contrast enhancement kinetics, we fitted an asymmetric generalised logistic function as regression function multiplied with an exponential term to the characteristic kinetic curve:

$$ C\left(t,G,\alpha, \tau, {t}_{1/2},\beta, k\right)=G\cdotp \left(1-\frac{1}{{\left(1+\left({2}^{\alpha }-1\right)\cdotp \exp \left(\frac{1}{\tau}\cdotp \left(t-{t}_{1/2}\right)\right)\right)}^{1/\alpha }}\right)\cdotp \exp \left(\beta \cdotp {t}^k\right) $$

where *G* defines the scaling, *α* the asymmetry parameter, *τ* the steepness, and *t*_1/2_ the time of half maximum of the sigmoid function; *k* defines the terminal slope and *β* scaling factor of the exponential term (Additional file 1: Figure S1). We used the parameters *α*, *τ*, *β*, and *k* as features (*f*_lkinetic_). In addition, we computed summary measures of the curve within a 7-min interval, beginning at start of contrast enhancement: area under the curve (*AuC*), maximum enhancement (*C*_max_), time to maximum enhancement (*T*_max_), time to half maximum enhancement (*T*_*1/2*_), and maximum analytical derivative $\frac{\delta C}{\delta t}$ of the regression function *C(t)* (*MDER*).

To obtain textural features, **f**_l-texture*,*_ we used a volumetric texture analysis approach based on grey-level co-occurrence matrix (GLCM) and Haralick texture features [17, 18]. We computed the GLCM with 128 Gy-value bins and 26 neighbours within the lesion and used its 13 s-order statistics [17]. **f**_l-tex-pre_, **f**_l-tex-peak_, and **f**_l-tex-post_ contained the Haralick features obtained from the **I**_dce-pre_, **I**_dce-peak_, and **I**_dce-post_ intensity values, respectively.

In addition to the spatial texture analysis, we used a novel temporal texture analysis inspired by the works of Agner et al. [19] and Woods et al. [20]. With this analysis, we characterised the temporal properties of contrast uptake within a lesion, *e.g*., homogeneity of contrast uptake. To compute the GLCMs, we considered voxel pairs at the same spatial position **x** but at different time points in the contrast enhancement. We computed the Haralick features from pixel pairs from (**I**_dce-pre_, **I**_dce-peak_), (**I**_dce-pre_, **I**_dce-post_), and (**I**_dce-peak_, **I**_dce-post_), resulting in the feature vectors **f**_l-tex-peak/pre_, **f**_l-tex-post/pre_, and **f**_l-tex-post/peak_.

To obtain morphological feature candidates, *f*_lmorph_, we used shape descriptors, as utilised previously in the literature [19, 21, 22]. Definitions of the shape descriptors are given in Additional file 1: Table S1.

### Evaluation of lesion segmentation and classification

To evaluate lesion segmentation, we performed experiments in a leave-one-out cross-validation (LOOCV) fashion, training the segmentation algorithm and feature rankings on all but one example, and applying it to the remaining example not included in the training. The quality of the segmentation was measured on a pixel level by comparing the predicted segmentation with the manually annotated data using Dice similarity coefficient (DSC) [23] as a similarity measure and sensitivity (true-positive rate) describing the probability of detection. As RF provide probabilities, we determined the RF threshold as the one that maximises DSC on the training set. Overall performance was obtained by computing the mean of all test DSC scores.

To evaluate lesion classification, we classified lesions into the two classes: benign and malignant. Evaluation was performed in an LOOCV fashion for both ranking the features and determining accuracy. Accuracy was reported as receiver operating characteristic (ROC) area under the curve (AUC) and sensitivity/specificity. The RF threshold was chosen within the training set as the one maximising the *F*_1_ score, which is the harmonic mean of precision and sensitivity. All experiments were repeated 20 times, and averages for AUC and sensitivity/specificity are reported. To study the impact of segmentation accuracy on classification, we performed classification on both manually delineated lesions and automatically segmented lesions.

In a post-processing step, false-positive blobs were removed by computing connected-components from the segmentations using a six-neighbourhood, and only blobs that partially overlapped with the manual annotation were selected. This step mimics the manual selection of a suspicious region that a radiologist wants to investigate further. For the two benign cases where the lesion was not detected, manual segmentation was used instead of the automatic segmentation. This post-processing step allowed us to evaluate classification accuracy independent of the segmentation performance.

### Evaluation of feature contribution

We then evaluated the contribution of features collected across the mpI data and ranked their contribution to segmentation and classification based on two measures: (1) RF Gini importance (GI) [10] and (2) minimum-redundancy-maximum-relevance (mRMR) [24]. The GI measures the average amount of information gain using the Gini index splitting criterion during RF training and ranks the contribution of each feature as part of a multivariate pattern. If features are redundant but informative, it ranks all of them highly [25]; the mRMR provides a ranking based on *relevance* and *redundancy* of the features. Then, we successively increased the number of features for training and validation, beginning with the top-ranked feature, and measured the performance of each model, thus allowing us to assess the contribution of each individual feature in a multimodal, multiparametric setup. In addition, the benefits of multiparametric and multimodal features were evaluated by training models using only DCE-MRI features and combined DCE-MRI, DWI, and/or ^18^F-FDG PET features.

## Results

### Lesion segmentation

**Fig. 4:**
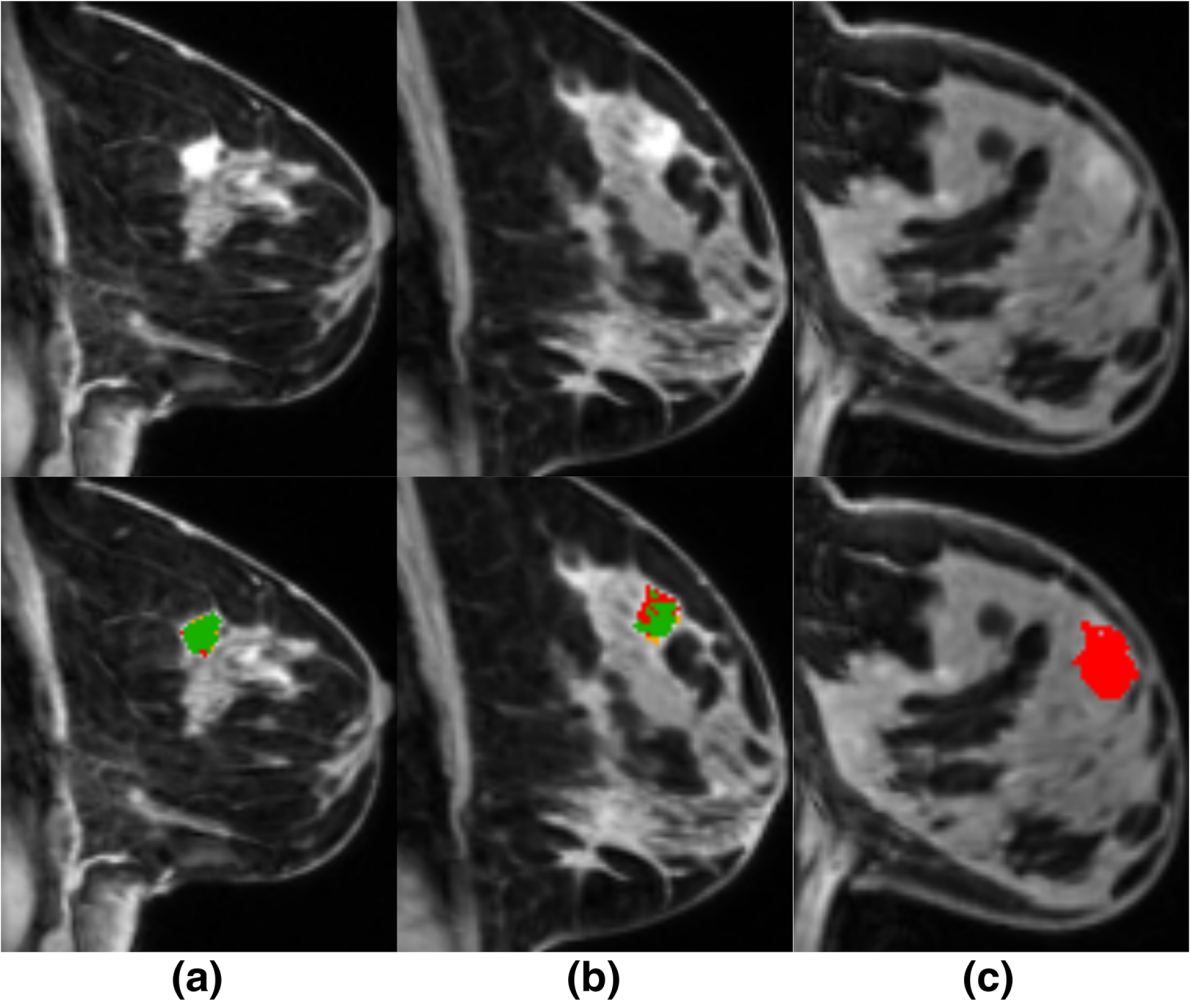
Segmentation results for the (**a**) best, (**b**) median, and (**c**) worst case according to the DSC score. The green colour indicates true-positive voxels, the yellow colour false-positive voxels, and the red colour false-negative voxels. Top row shows I_dce-post_

We report in Table 3 and illustrate in Additional file 1: Figure S2 the performance of the models showing the highest DSC for Gini importance and mRMR feature selection with and without multiparametric features. The model with mRMR feature selection and the top eight features showed a mean/median DSC of 0.665/0.757. Here, DSC benefited from multiparametric features, showing a reduced mean DSC of 0.601 without DWI, 0.618 without PET, and 0.584 with only DCE-MRI features. The model with GI feature selection showed a lower performance with a DSC of 0.607 compared with the model with mRMR feature selection. Here, DSC also benefitted from multiparametric features, showing a reduced DSC of 0.577 with only DCE-MRI features. The improvement in segmentation accuracy for multiparametric features mainly resulted from reducing false-positive cases, such as vessels and enhancing parenchymal areas. Overall, for this dataset, we had a detection rate of 22/22 (100%) for malignant lesions and of 10/12 (83.3%) for benign lesions. As shown in Fig. 4, the missed benign lesions had a very low contrast uptake and thus were missed by the prediction models.

**Table 3:** Automatic segmentation performance in terms of DSC and sensitivity

Features	DSC mean ± SD/median ± IQR	Sensitivity mean ± SD/median ± IQR	Number of features
GI	0.607 ± 0.238/0.691 ± 0.255	0.661 ± 0.234/0.721 ± 0.263	2
GI without PET	0.608 ± 0.281/0.722 ± 0.419	0.739 ± 0.290/**0.876** ± 0.238	11
GI without DWI	0.573 ± 0.290/0.708 ± 0.445	0.669 ± 0.324/0.825 ± 0.445	8
GI without DWI, without PET	0.577 ± 0.277/0.665 ± 0.419	0.658 ± 0.343/0.813 ± 0.597	25
mRMR	**0.665** ± 0.236/**0.757** ± 0.189	0.743 ± 0.267/0.836 ± 0.269	8
mRMR without PET	0.618 ± 0.272/0.749 ± 0.413	**0.748 ± ** 0.277/0.873 ± 0.307	7
mRMR without DWI	0.601 ± 0.268/0.701 ± 0.377	0.686 ± 0.328/0.801 ± 0.538	7
mRMR without DWI, without PET	0.584 ± 0.300/0.710 ± 0.393	0.613 ± 0.338/0.784 ± 0.496	8

*DSC* Dice similarity coefficient, *DWI* diffusion-weighted imaging, *IQR* interquartile range, *GI* Gini Importance, *mRMR* minimum-redundancy-maximum-relevance, *PET* positron emission tomography, *SD* standard deviation. Values presented in bold are the highest values

**Fig. 5:**
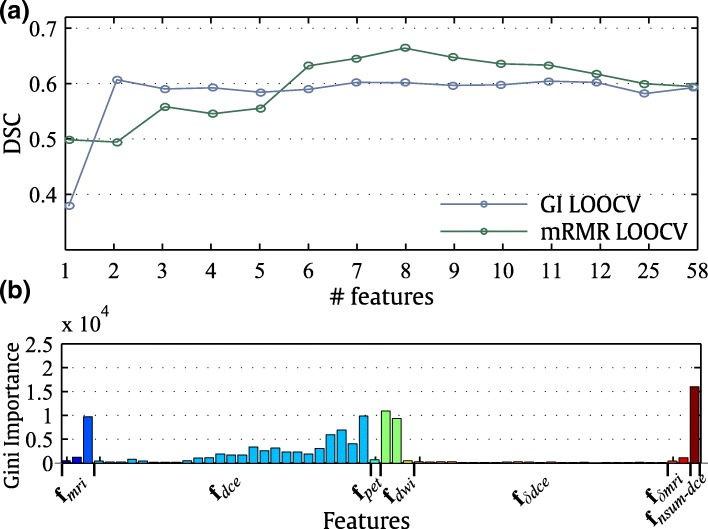
Feature ranking and its influence on segmentation performance. **a** The mean DSC, using a successively increasing number of top-ranked features according to RF GI and mRMR ranking. **b** GI feature ranking of the segmentation features. The four top-ranked features are labelled in the figure

The performance of the GI and mRMR feature selection models with an increasing number of highest-ranked features is shown in Fig. 5a. The performance of the GI feature selection model peaked at only three features whereas the performance of the mRMR feature selection model peaked at six features. Table 4 shows the ranking of the features according to GI and mRMR. Both algorithms ranked **f**_dwi_, **f**_nsum-dce_, and **I**_dce-post_ highly. However, mRMR tended to pick more varied features than GI, where GI selected six potentially correlated features from **f**_dce_ as part of the top 10 features. The features capturing changes in the contrast, **f**_δdce_ and **f**_δmri_, received a lower ranking in GI (see also Fig. 5b) compared with mRMR.

**Table 4:** Top-ranked segmentation features according to Gini importance and minimum-redundancy-maximum-relevance

Rank	Gini importance	Minimum-redundancy-maximum-relevance
1	**f**_nsum-dce_	**I**_dce-post_
2	**f**_dwi_ # 1	**f**_δdce_ #16
3	**f**_dce_ # 25	**f**_dwi_ # 2
4	**I**_dce-post_	**f**_pet_
5	**f**_dwi_ # 2	**f**_nsum-dce_
6	**f**_dce_ # 23	**f**_dwi_ #1
7	**f**_dce_ # 22	**f**_δmri_ #1
8	**f**_dce_ # 24	**f**_δdce_ #23
9	**f**_dce_ # 15	**f**_δmri_ #2
10	**f**_dce_ # 17	**f**_δdce_ #20

Numbers next to each feature (#) indicate the frame index in the dynamic contrast-enhanced magnetic resonance imaging. *DSC* Dice similarity coefficient. See text for the abbreviations subscripted after **f** or **I**

### Lesion classification

In Table 5, we list the results for the models showing the highest ROC AUC score after GI and mRMR feature selection. Overall, for manually annotated lesions, mRMR feature selection yielded the highest AUC (0.978) using only two features, with a sensitivity of 94.6% and specificity of 93.6% for identifying malignant lesions. When automatic segmentation was used, the highest ROC AUC was 0.861 including only three DCE-MRI features. mRMR feature selection showed a better AUC performance than GI, both for manual annotation (0.978 *versus* 0.949) and automatic segmentation (0.861 *versus* 0.771).

**Table 5:** Classification results for differentiation of malignant and benign lesions for manually annotated lesions and automatic segmented lesions using automatic feature selection

Feature selection method	Manual annotation			Automatic segmentation		
	AUC (mean ± SD)	Sensitivity/specificity	Number of features	AUC (mean ± SD)	Sensitivity/specificity	Number of features
GI LOOCV	0.949 ± 0.019	0.920/0.868	4	0.771 ± 0.040	0.961/0.482	100
GI LOOCV without PET	0.946 ± 0.002	0.924/0.859	4	0.771 ± 0.040	0.972/0.486	75
GI LOOCV without DWI	0.949 ± 0.015	0.915/0.873	4	0.754 ± 0.035	**0.983**/0.427	75
GI LOOCV without DWI, without PET	0.944 ± 0.018	0.922/0.868	4	0.755 ± 0.033	0.976/0.409	75
mRMR LOOCV	**0.978** ± 0.008	0.946/0.936	2	0.858 ± 0.013	0.941**/0.773**	3
mRMR LOOCV w/o PET	0.975 ± 0.010	**0.957**/0.918	2	0.856 ± 0.018	0.948/0.736	3
mRMR LOOCV w/o DWI	0.977 ± 0.006	0.954/**0.950**	2	0.857 ± 0.017	0.943/0.745	3
mRMR LOOCV w/o DWI, PET	0.973 ± 0.010	0.950/0.927	2	**0.861** ± 0.009	0.941/0.755	3

*AUC* area under the curve at receiver operating characteristic analysis, *DCE-MRI* dynamic contrast-enhanced magnetic resonance imaging, *DWI* diffusion-weighted imaging, *GI* Gini importance, *LOOCV* leave-one-out cross-validation, *mRMR* minimum-redundancy-maximum-relevance, *PET* positron emission tomography, SD standard deviation. Values presented in bold are the highest values

**Fig. 6:**
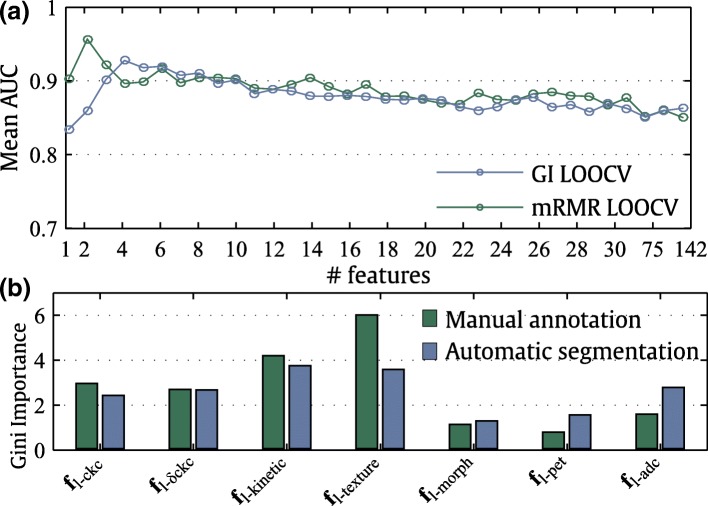
Feature ranking and its influence on classification performance. **a** Mean ROC-AUC using an increasing number of top-ranked features according to GI and mRMR ranking. **b** GI ranking showing the top-ranked classification features of each feature-group, computed from manual annotations (green) and automatic segmentations (blue)

The performance of the GI and mRMR feature selection models with an increasing number of highest-ranked features is shown in Fig. 6a. The mRMR feature selection model peaked at only two features whereas the GI feature selection model peaked at four features, with a subsequent decrease in AuC performance. A closer look at the ranking of the features (Table 6 and Fig. 6b) indicates that features from the pool of kinetic (**f**_l-kinetic_) and textural (**f**_l-texture_) features were top-ranked by GI and mRMR models. Morphologic (**f**_l-morph_) and PET (**f**_l-pet_) features received a low ranking by GI and mRMR models. The DWI ADC feature (**f**_l-adc_) was ranked as an important feature by GI in automatic segmentation only.

**Table 6:** The ten top-ranked classification features according to Gini importance and minimum-redundancy-maximum-relevance

Rank	Manual annotation		Automatic segmentation	
	Gini importance	Minimum-redundancy-maximum-relevance	Gini importance	Minimum-redundancy-maximum-relevance
1	**f**_l-tex-post/peak_ entropy	**f**_l-tex-post/peak_ energy	**f**_l-kinetic_ *T*_1/2_	**f**_l-tex-post/peak_ energy
2	**f**_l-tex-post/peak_ energy	**f**_l-δckc_ # 11	**f**_l-tex-post/peak_ energy	**f**_l-δckc_ #10
3	**f**_l-kinetic_ *T*_1/2_	**f**_l-δckc_ # 8	**f**_l-kinetic_ MDER	**f**_l-tex-post/pre_ sum average
4	**f**_l-ckc_ #11	**f**_l-kinetic_ MDER	**f**_l-adc_	**f**_l-tex-post/pre_ homogeneity
5	**f**_l-ckc_ #9	**f**_l-tex-post/pre_ homogeneity	**f**_l-δckc_ #18	**f**_l-δckc_ #20
6	**f**_l-tex-post_ information measure 1	**f**_l-tex-post/peak_ correlation	**f**_l-ckc_ #11	**f**_l-tex-post/peak_ information measure 2
7	**f**_l-δckc_ #14	**f**_l-tex-post/peak_ entropy	**f**_l-δckc_ #21	**f**_l-δckc_ #21
8	**f**_l-tex-post_ information measure 2	**f**_l-kinetic_ *T*_1/2_	**f**_l-δckc_ #14	**f**_l-kinetic_ *β*
9	**f**_l-δckc_ #11	**f**_l-tex-post/pre_ dva	**f**_l-tex-post/pre_ dva	**f**_l-tex-peak/pre_ homogeneity
10	**f**_l-tex-peak_ entropy	**f**_l-tex-post/pre_ energy	**f**_l-δckc_ #10	**f**_l-tex-post_ sum variance

*dva* difference variance, *MDER* maximum derivative of kinetic regression function

## Discussion

We present a novel data-driven combined breast lesion segmentation and classification system for mpI data with combined ^18^F-FDG-PET/MRI. This system automatically detects and segments potentially cancerous regions and classifies lesions as benign or malignant. Our results showed that automatic lesion segmentation was accurate and improved with information from all modalities, but even a small number of features were sufficient to achieve the reported maximum accuracy. On the other hand, our results showed that lesion classification largely drew on information from DCE-MRI, without benefitting from information from other modalities and parameters. The results are consistent with previous findings but add insights into the feasibility of a completely automated lesion segmentation and of classification from mpI data. The results were obtained by quantifying the information captured across multimodal mpI data and features, enabling the assessment of imaging protocols in this context.

Using combined mpI based on DCE-MRI, DWI, and ^18^F-FDG-PET in a CADe or CADx system is a novel promising approach for improving diagnostic accuracy [26]. Previously, CADe and CADx systems have been proposed for digital mammography to increase the rather moderate sensitivity [27] and to help in classifying lesions as benign or malignant [28]. Semi-automatic methods have been proposed for classifying each pixel as cancerous or non-cancerous using fuzzy c-means clustering [29] or Markov random field-based clustering of the time-series [30]. Moreover, methods designed to outline lesions using the *active contour* framework (*i.e.*, autonomously and adaptive search of object contours based on image features and user interaction) have also been presented [31, 32]. Automatic segmentation methods, which may also be seen as CADe systems, have been proposed using machine-learning approaches based on intensity and textural features (co-occurrence, run-length) [20, 33–35]. Recently, an automated localisation of breast cancer lesions based on DCE-MRI was proposed by Gubern-Mérida et al. [36]. Multimodal approaches combining several modalities have been reported for PET/CT breast images: Han et al. [37] segmented lesions by applying a graph-based Markov random field method on a combined PET/CT image, taking advantage from both the high spatial resolution of CT and the functional information of PET. Lastly, several CADx methods that classify breast lesions as benign or malignant by exploring the DCE-MRI data have been proposed using morphology [38], lesion texture [39], contrast enhancement [16, 40], a combination of morphology and contrast enhancement [41], or a combination of morphology and texture [19, 21, 31, 42, 43]. State-of-the-art DCE-MRI CADx methods have been reported using various performance metrics, different datasets (*e.g.*, malignant cases only), and differing aims (*i.e.*, segmentation *versus* detection).

Using our system, we detected all malignant cases and missed two benign lesions. Detected lesions were classified as malignant with a sensitivity of 95%. Using texture features, Woods et al. [20] and Yao et al. [35] previously reported an ROC-AUC of 0.999 and 0.984, respectively. However, Woods et al. performed the evaluation on the same subjects as used in training, and both these studies were conducted in a small set of malignant lesions only. Twellmann et al. [33] reported a ROC-AUC of 0.99 for lesion detection using LOOCV and DCE-MRI information. Vignati et al. [34] reported the performance of a fully automated system as a detection rate of 0.89 and a sensitivity of 0.98 at four false-positive cases per breast. In their study, the performance measure did not include false-positive areas. Gubern-Mérida et al. [36] used an automated method and achieved a sensitivity of 89% at four false-positive per normal case. As normal cases, they included patients with a BI-RADS rating of 1 or 2, who were healthy subjects with benign findings.

For the task of automatic lesion segmentation, our study showed that mpI is beneficial, as evidenced by the increase of the DSC from 0.584 to 0.665. The high ranking of DWI features in both GI and mRMR feature selection models indicates that the addition DWI to DCE-MRI is especially beneficial in segmentation. We also found that lesion segmentation benefitted from the addition of PET, although the benefit was to a lesser extent than that of DWI. When both DWI and PET were added, the DSC was further improved; thus, our results suggest that PET has a complementary relationship with DWI. Interestingly, features describing the change of contrast between time-steps (**f**_δdce_ and **f**_δmri_) received a good ranking in the mRMR feature selection model overall but a low ranking in the GI feature selection model. A likely reason is that while they contribute less information than the higher-ranked GI features, their contribution is orthogonal to the higher-ranked features. In our study, mRMR as a feature selection model provided slightly better results than GI. The moderate mean DSC score for lesion segmentation results from several reasons. First, the two undetected benign lesions exhibited very low contrast enhancement with a DSC of 0, leading to a drop in the mean value. However, we kept these two benign cases in the dataset to evaluate whether additional parameters may allow the system to segment these challenging cases, which was not the case as reported. Second, additional areas of contrast uptake, such as vessels and enhancing parenchymal tissues, resulted in an increased false-positive rate. While DWI and ^18^F-FDG-PET image modalities increased automatic segmentation accuracy, mainly by reducing the false-positive cases, lesions with low contrast uptake could not be detected automatically. As good segmentation is important for the accurate classification of a lesion, we aim to improve the segmentation performance, *e.g*., by introducing heuristics that filter false-positive cases in a post-processing step in a future study, as proposed for instance by Vignati et al. [34] and Gubern-Mérida et al. [36] where morphologic and kinetic descriptors were used in a second step.

In our study, a high accuracy in lesion classification was achieved for both expert and automatic segmentation. However, the highest accuracy was achieved with manual segmentation and mRMR feature selection from DCE imaging data. Top-ranked features largely overlapped between GI and mRMR feature selection models; the exception was that **f**_l-adc_ was ranked highly by the GI feature selection model following only automatic segmentation. While the addition of DWI and ^18^F-FDG-PET to DCE-MRI was beneficial overall for lesion segmentation, lesion classification only improved slightly with these two modalities for GI feature selection following manual segmentation. Lesion classification for mRMR features selection was best without these two modalities. **f**_l-pet_ was lowly ranked, consistent with recent findings by Magometschnigg et al. [44] that indicate that quantitative ^18^F-FDG-PET values are not helpful for breast cancer classification. On the other hand, the kinetic feature **f**_l-kinetic_ received a high GI as well as high mRMR ranking. Textural features were top-ranked, mostly from **f**_l-tex-post/peak_. The top-ranked feature, GLCM energy, measures the uniformity of lesion texture, reflecting the uniformity of contrast-enhancement within the lesion during a later stage. The morphologic feature **f**_l-morph_ scored very low, although they are an integral part of the BI- RADS® lexicon for lesion classification, being discriminative features for clinical diagnosis, as shown by Pinker-Domenig et al. [45]. This suggests that binary segmentation and shape descriptors are not precise enough to describe the shape and margin of the lesion and feature extraction from a soft-margin around the hard segmentation border (*e.g.*, textural features) may better capture the BI-RADS margin descriptors (circumscribed, non-circumscribed, irregular, spiculated). Alternatively, digital mammography or digital breast tomosynthesis may be used as an additional higher resolution modality to assess the morphology of the lesion more accurately. To summarise, mRMR slightly outperformed GI as a feature selection method for breast lesion classification. Novel DCE-MRI features that describe the kinetics and spatio-temporal texture of the contrast uptake were highly predictive for the classification of benign and malignant lesions, whereas DWI and PET did not provide additional information. Whereas we used data from separate MRI and PET/CT scanners, the methods, results, and findings can be directly transferred to images obtained at combined PET/MRI scanners, as the CT information was used for alignment only and was not part of the decision models.

One limitation of the study is that only subjects with suspicious findings on mammography or breast ultrasonography were included. As a consequence, an assessment of false-positive cases in healthy subjects was not possible. However, the majority of tissue in the breast consists of healthy tissue, on which the classifier was trained, and was classified as healthy tissue in our study. A second limitation is the small number of subjects. Even though cross-validation allowed us to estimate the generalisation of the model to some degree, statistical significance can only be obtained from a larger cohort. Thus, we aim to confirm our preliminary findings on a larger number of patients in a future study.

In conclusion, we used an entirely data-driven approach in combination with the assessment of the contribution of individual imaging parameters to provide a means for in-depth understanding of the multivariate information, where redundancies and relationships between imaging data are not obvious. This is essential for further clinical exploitation of imaging parameters. It enables designing of feasible imaging paradigms constructed from a possibly reduced subset of acquisition sequences. Furthermore, in the context of disease mechanisms, the data-driven model could serve as a means for hypothesis generation.

## Additional file

Additional file 1: **Figure S1.** Illustration of the influence of logistic model parameters on curve, and the model fitted to a CKC. From left to right: α defines the asymmetry of the logistic model, τ the steepness of the curve and k influences the terminal slope. The regression curve fitted to a given CKC for a malignant (blue) and a benign lesion (green). **Figure S2.** Boxplot of automatic segmentation performance in terms of Dice similarity coefficient (DSC). DWI, diffusion-weighted imaging; GI, Gini Importance; mRMR, minimum-Redundancy-Maximum-Relevance; PET, positron emission tomography; w/o, without. (DOCX 219 kb)

## Abbreviations

^18^F-FDG: ^18^F-fluorodeoxyglucose
ADC: Apparent diffusion coefficient
BI-RADS: Breast Imaging-Reporting and Data System
CADe: Computer-aided detection
CADx: Computer-aided diagnosis
CT: Computed tomography
DCE-MRI: Contrast-enhanced magnetic resonance imaging
DSC: Dice similarity coefficient
DWI: Diffusion-weighted imaging
FLASH: Fast low angle shot
GI: Gini importance
GLCM: Grey-level co-occurrence matrix
LOOCV: Leave-one-out cross-validation
mpI: Multiparametric imaging
mRMR: Minimum-redundancy-maximum-relevance
PET: Positron emission tomography
RF: Random forest
ROC: Receiver operating characteristic
VIBE: Volumetric interpolated breath-hold-examination
