# Supplementary material for: Automatic segmentation and classification of breast lesions through identification of informative multiparametric PET/MRI features
Source: Eur Radiol Exp. 2019 Apr 27;3:18. doi: 10.1186/s41747-019-0096-3 (PMC6486931; doi:10.1186/s41747-019-0096-3)
Supplement: Supplementary file 1 — Figure S1. Illustration of the influence of logistic model parameters on curve, and the model fitted to a CKC. From left to right: α defines the asymmetry of the logistic model, τ the steepness of the curve and k influences the terminal slope. The regression curve fitted to a given CKC for a malignant (blue) and a benign lesion (green). Figure S2. Boxplot of automatic segmentation performance in terms of Dice similarity coefficient (DSC). DWI, diffusion-weighted imaging; GI, Gini Importance; mRMR, minimum-Redundancy-Maximum-Relevance; PET, positron emission tomography; w/o, without. (DOCX 219 kb) [file 41747_2019_96_MOESM1_ESM.docx]

Additional file 1: Figure S1 Illustration of the influence of logistic model parameters on curve, and the model fitted to a CKC. From left to right: α defines the asymmetry of the logistic model, τ the steepness of the curve and *k* influences the terminal slope. The regression curve fitted to a given CKC for a malignant (blue) and a benign lesion (green).


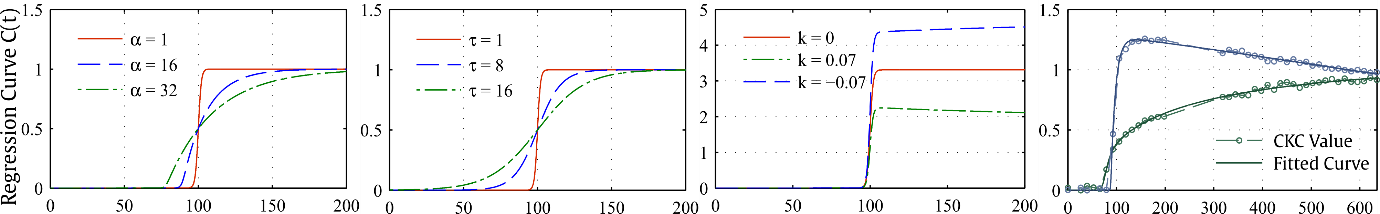


Figure S2 Boxplot of automatic segmentation performance in terms of Dice similarity coefficient (DSC). DWI, diffusion-weighted imaging; GI, Gini Importance; mRMR, minimum-Redundancy-Maximum-Relevance; PET, positron emission tomography; w/o, without

*
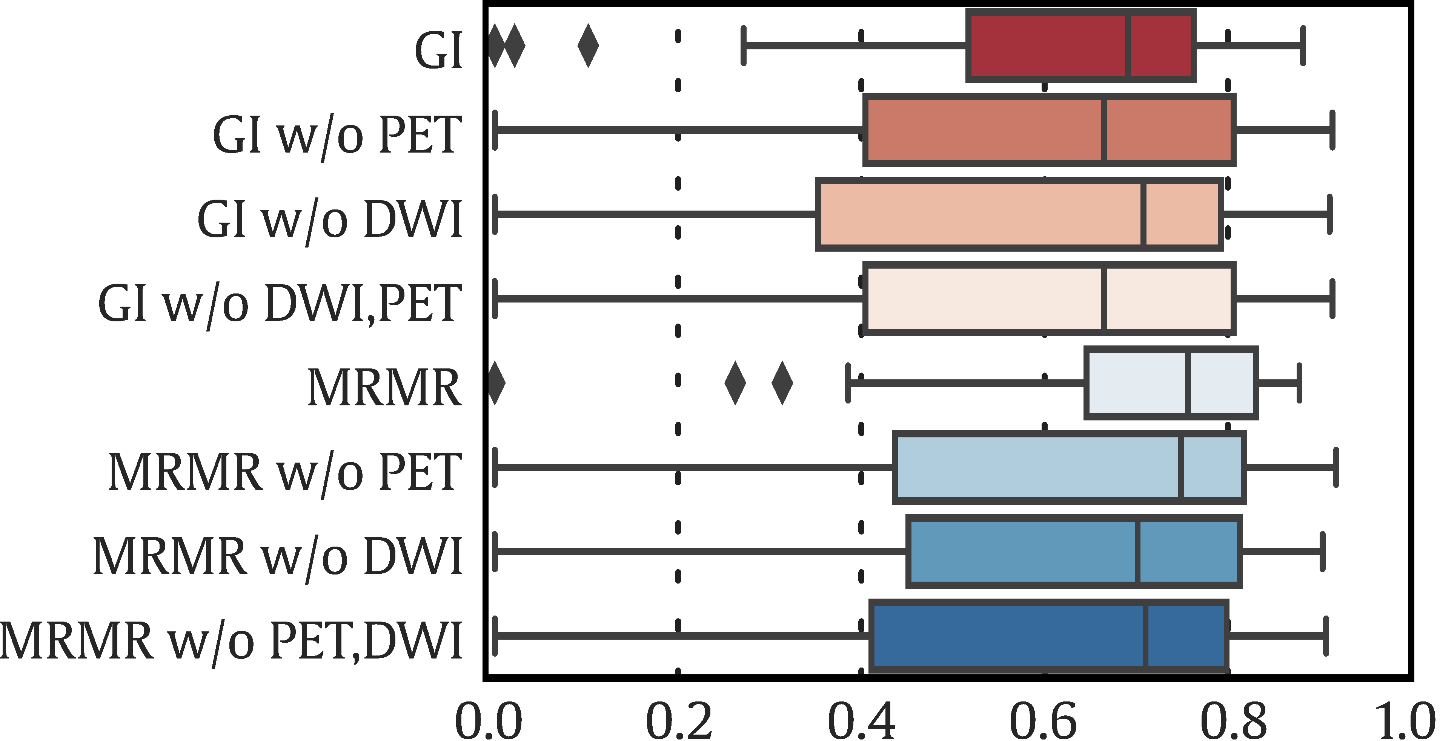
*

Table S1 Definitions of morphologic features.

| **Morphological feature** | **Definition** | **Note** |
| --- | --- | --- |
| Number of voxels in the lesion ($n$) = Volume of lesion ($V$) | $n=\vert\mathbf{x}_{l}\vert$ | Number of voxels and volume were the same in this case since a voxel had a volume of 1 mm${}^{3}$. |
| Centroid ($\bar{c}$) | $\bar{c}=\frac{1}{n}\sum_{n} \mathbf{x}_{i}$, with $\mathbf{x}_{i}\in\mathbf{x}_{l}$ |  |
| Maximum radial distance ($r$) | $r=\max_{\mathbf{x}_{i}\in\mathbf{x}_{l}}(\vert\vert\mathbf{x}_{i}-\bar{c}\vert\vert)$ |  |
| Normalized radial length ($nrl$) | $nrl_{i}=\frac{\vert\vert\mathbf{x}_{i}-\bar{c}\vert\vert}{r}$, with $\mathbf{x}_{i}\in\mathbf{x}_{l}$ |  |
| Area of enclosing surface ($A$) | $\vert voxel surface faces\vert$ |  |
| Volume Overlap Ratio ($VOR$) | $\frac{V}{4/3\pi r^{3}}$ | Ratio of lesion volume to circumscribing sphere. |
| Elliptic Volume Overlap Ratio ($EllVOR$) | $\frac{V}{4/3\pi a b c}$ | Ratio of lesion volume to inertia ellipsoid. $a,b,c$...ellipsoid radii. The inertia ellipsoid is obtained by PCA of $\mathbf{x}_{l}$. |
| Discrete Compactness ($C_{d}$) ^52^ | $\frac{n-A/6}{n-(\sqrt[3]{n})^{2}}$ |  |
| Irregularity ($Irr$) | $1-\frac{\pi*d_{e}^{2}}{A}$, with $d_{e}=2\sqrt[3]{\frac{3V}{4\pi}}$ | Deviation of lesion surface $A$ from a sphere surface with the same volume. |
| Sphericity ($S$) | $\frac{\min_{\mathbf{x}_{i}\in\mathbf{x}_{l}}(\vert\vert\mathbf{x}_{i}-\bar{c}\vert\vert)}{r}$ | Ratio of inscribing sphere radius to circumscribing sphere. |
| NRL mean ($\mu_{nrl}$) | $1/n\sum_{n} nrl_{i}$ |  |
| NRL variance ($\sigma_{nrl}^{2}$) | $1/n\sum_{n} (nrl_{i}-\mu_{nrl})^{2}$ |  |
| NRL sphericity ($S_{nrl}$) | $\frac{\mu_{nrl_{i}}}{\sigma_{nrl}^{2}}$ |  |
